# Tissue-specific effects of bacterial PncA overexpression on NAD^+^ metabolism and aging in mice: implications for tissue-specific aging interventions

**DOI:** 10.3389/fragi.2025.1546017

**Published:** 2025-04-28

**Authors:** Fengjiao Huo, Meili Zhao, Yue Liu, Shuyao Lv, Shengyu Feng, Liuling Guo, Nan Wang, Shuaishuai Zhang, Qing Liu, Taotao Mi, Hao Wang, Jian-Kang Zhu, Hailiang Liu

**Affiliations:** ^1^ State Key Laboratory of Cardiology and Medical Innovation Center, Institute for Regenerative Medicine, Shanghai East Hospital, School of Medicine, Tongji University, Shanghai, China; ^2^ Institute of Advanced Biotechnology and School of Medicine, Southern University of Science and Technology, Shenzhen, China; ^3^ Key Laboratory of Xinjiang Phytomedicine Resource and Utilization of Ministry of Education, College of Life Sciences, Shihezi University, Shihezi, China

**Keywords:** aging, nicotinamide adenine dinucleotide, nicotinamide, nicotinic acid, pncA

## Abstract

**Background:**

As a critical molecule in biological systems, nicotinamide adenine dinucleotide (NAD^+^) influences the aging of mammals. Therefore, regulation of NAD^+^ synthesis and degradation may slow aging and mitigate related diseases.

**Results:**

This study investigated how mammalian tissues rely on different NAD^+^ synthesis pathways and prefer specific NAD^+^ precursors. Overexpressing the bacterial nicotinamidase PncA in mice increased NAD^+^ levels in the liver and kidneys but decreased levels in the heart and hippocampus. In aged mice (25 months old), this overexpression delayed aging indicators by boosting NAD^+^ levels in the liver and kidneys, indicating potential for PncA to improve age-related decline in these tissues. However, in younger mice (4 months old), PncA overexpression accelerates the senescence of cardiac cells, resulting in a reduction of NAD + levels, increased aging markers, and cognitive decline. These disparate results underscore the necessity of a nuanced, tissue-specific perspective when contemplating the use of NAD^+^ precursor supplementation as a means of addressing aging.

**Conclusion:**

Our study highlights the complexity of NAD^+^ metabolism and its effects on aging in various tissues. It suggests personalized interventions for aging and age-related diseases by showing how different tissues respond to NAD^+^ precursor manipulation, emphasizing the importance of targeted strategies for optimal therapeutic results with minimal side effects.

## Graphical Abstract

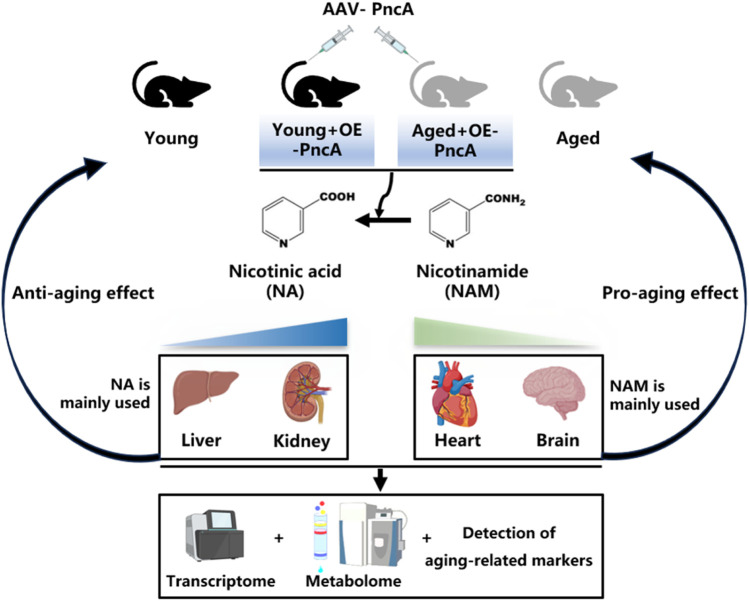

## Highlights

1. Overexpressing the bacterial nicotinamidase (PncA) in mice raised NAD^+^ levels in the liver and kidneys, but lowered levels in the heart and hippocampus.
2. The impact of PncA on NAD^+^ levels exhibits tissue-specificity.
3. PncA overexpression delayed aging markers in the liver and kidneys but accelerated aging and cognitive decline in the heart and hippocampus.

## Introduction

Nicotinamide adenine dinucleotide (NAD^+^) is one of the most essential small molecules in biology, playing a pivotal role in various cellular processes, including energy metabolism, DNA repair, and cell signaling (Rajman et al., 2018; Covarrubias et al., 2021; Imai and Guarente, 2014). As mammals age, a decline in NAD^+^ levels has been observed, correlating to the onset of age-related physiological decline and diseases (Verdin, 2015). This connection has focused significant attention on NAD^+^ metabolism as a potential target to delay aging and treat age-associated disorders (Aman et al., 2018; Katsyuba and Auwerx, 2017; Wilson et al., 2023).

The primary NAD^+^ synthesis pathways include *de novo*, Preiss–Handler, and salvage pathways (Yoshino et al., 2018). The *de novo* pathway synthesizes NAD^+^ from tryptophan, which is a complex, multistep process (Katsyuba et al., 2018). The second pathway involves conversion of nicotinic acid (NA) into NAD^+^, which is prominent in tissues with access to dietary sources of NA (Bogan and Brenner, 2008). The salvage pathway is the most prominent pathway in mammals, which recycles nicotinamide (NAM) back into NAD^+^. NAM serves as a substrate in reactions involving NAD^+^-consuming enzymes, such as sirtuins, ADP-ribosyltransferases, and poly (ADP-ribose) polymerases (Imai and Guarente, 2014; Aravind et al., 2015; Cohen, 2020). These enzymes use NAD^+^ in processes such as deacetylation, ADP-ribosylation, and DNA repair, leading to NAD^+^ depletion and NAM release. The activity of these enzymes influences the cellular NAD^+^ level, and the resultant NAM acts as a feedback inhibitor, particularly for sirtuins, playing an important role in cellular metabolic regulation (Hwang and Song, 2017; Lee et al., 2019).

NAD^+^ biosynthesis varies across tissues in mammals, suggesting tissue-specific dependency on NAD^+^ precursors and metabolic pathways (Bogan and Brenner, 2008; Walzik et al., 2023; McReynolds et al., 2021; Zou et al., 2020). However, direct evidence that highlights these differences remains insufficient. PncA, an enzyme that catalyzes conversion of NAM to NA, has emerged as a critical player in modulating the NAD^+^ level (Feng et al., 2023; Shats et al., 2020). Our study focused on overexpression of PncA in mice, and its subsequent effects on NAD^+^ metabolism and aging in various tissues.

This study demonstrates that the intestinal microbiota converts NAM into NA via the nicotinamide enzyme PncA, thereby activating the host’s Preiss-Handler pathway to synthesize NAD^+^. This discovery unveils a novel mechanism of microbial-host synergistic regulation of NAD^+^ metabolism (Shats et al., 2020). It broadens the conventional understanding of NAD^+^ synthesis pathways, which traditionally focus solely on NAMPT and NAPRT, and suggests that interventions targeting the microbiota could enhance the tissue-specific effects of NAD^+^ supplementation (e.g., NAM). Such interventions could potentially improve liver metabolism or intestinal barrier function, offering tissue-specific regulatory strategies for anti-aging therapies (Shats et al., 2020; Chini et al., 2024). Additionally, the study indicates that microbiota metabolism may affect the efficacy of conventional NAD^+^ inhibitors (NAMPT inhibitors), underscoring the necessity for a comprehensive evaluation of host-microbial interaction networks in aging interventions (Shats et al., 2020; Li et al., 2024).

## Materials and methods

### Materials

Kits used in our research: AAV purification kit (ViraTrap™, BEIWO Co., Ltd, China), EnzyChrom™ NAD^+^/NADH^+^ Assay Kit (E2ND-100, BioAssay Systems, Hayward, United States), ammonia detection kit (LabAssay™ ammonia, Wako, Japan), DNA extraction kit (YDP304, TIANGEN, China), HiScript III All-in-one RT SuperMix (R333, Vzayme, China), Sirtuin 1 (SIRT-1) Activity Assay Kit (Elabscience Biotechnology, Wuhan, China). Reagents used in our research: Trizol reagent (15596026, Invitrogen, USA),qPCR Supermix (Q712, Vzayme, China), Lipofiter™ (HB-TRCF-1000, Hanbio Biotechnology, China).

### Mice

Wildtype female C57BL/6J mice were purchased from SLAC ANIMAL Inc (Shanghai, China). Mice were maintained under a 12 h light/dark cycle and fed a standard chow diet in the specific pathogen-free facility at the Laboratory Animal Research Center, Tongji University. For AAV infection, we used 8-month-old mice to elucidate the effect of PncA on the NAD^+^ level across various tissues in middle-aged mice. Administration of AAV9-PncA was performed by tail vein injection at a dose of 1 × 10^9^ genomic copies. Additionally, 4- and 25-month-old C57BL/6J mice were subjected to tail vein injection of 1 × 10^9^ AAV9-PncA to investigate the influence of PncA on the aging process in young and elderly mice, respectively. Two months following AAV administration, the mice were euthanized using pentobarbital. Subsequently, tissue samples were collected and rapidly frozen with liquid nitrogen, then stored at −80°C until further analysis.

The brain hemispheres were placed on an ice-cold glass dissection plate and orientated in the sagittal plane. The right hemispheres were placed in 4% paraformaldehyde for cryostat section ing and immunohistochemistry and stored at − 80°C until required.

### AAV construction

The production of adeno-associated virus (AAV) was conducted in 293T cells using a tri-plasmid transfection method, which included pAAV-RC, pHelper, and either shuttle plasmids containing the gene of interest or control plasmids. Prior to transfection, 293T cells were cultured in 100-mm dishes until they reached 80%–90% confluence. The transfection mixture consisted of 10 μg of pAAV-RC, 20 μg of pHelper, 10 μg of the shuttle plasmid, and 120 μL of LipofiterTM transfection reagent (HB-TRCF-1000, Hanbio Biotechnology Co., Ltd, Shanghai, China). Six hours after transfection, the growth medium was replenished with complete growth medium containing 10% fetal bovine serum (FBS). At 72 h post-transfection, cells containing AAV particles were detached using a cell scraper, collected in a 15-mL tube, and subjected to a brief centrifugation at 150 × g for 3 min. The purification of AAV from cells was carried out using ViraTrap™ (BEIWO Co., Ltd, Hangzhou, China) following the protocol provided in the kit.

### Nicotinamidase activity assay

Mouse tissues (50 mg) were homogenized in 1 mL PBS. The assay involved incubating 100 mM HEPES (pH 7.4), 500 μM NAM, and 20 μL tissue homogenate at 27°C. Addition of the tissue homogenate initiated the enzymatic reaction, and ammonia production was quantified after 30 min. NAM conversion in the tissue homogenate was assessed by measuring ammonia generation using a specialized assay kit (LabAssay™ ammonia, Wako, Japan) (Inokuma K. et al., 2018).

### RT-qPCR

RNA extraction from mouse tissues was performed using Trizol reagent (Invitrogen, Carlsbad, CA, USA). The isolated RNA (1 μg) was subjected to DNase treatment and underwent reverse transcription to generate cDNA. The resulting cDNA was diluted 10-fold and subjected to quantitative PCR. qPCR analysis was conducted using the LightCycler system (Roche Diagnostics GmbH, Rotkreuz, Switzerland) and qPCR Supermix (Vzayme, Nanjing, China) with specific primers (Supplementary Table S1). Each biological data point was based on the average of at least three technical replicates.

### Quantification of mitochondria

Mitochondrial numbers were determined by the mtDNA:nDNA ratio. The method for quantifying mtDNA was as previously described (Lagouge M. et al., 2006; Katsyuba E. et al., 2018), with some minor modifications. DNA was extracted from mouse tissues using a DNA extraction kit (# YDP304, Tiangen biotech, Beijing, China) and diluted to 10 ng/μl as a qPCR template. The nLPL gene was selected as the reference gene, and the mMito gene was used to quantify mitochondrial content. The following primer sequences were used: nLPL-F, GGA​TGG​ACG​GTA​AGA​GTG​ATT​C and nLPL-R, ATC​CAA​GGG​TAG​CAG​ACA​GGT; mMito-F, CTA​GAA​ACC​CCG​AAA​CCA​AA and mMito-R, CCA​GCT​ATC​ACC​AAG​CTC​GT.

### Quantification of NAD^+^

To quantify the levels of NAD^+^, the EnzyChrom™ NAD^+^/NADH^+^ Assay Kit (E2ND-100) from BioAssay Systems (Hayward, CA, USA) was employed. In brief, technical replicates of samples obtained from the same animal, each weighing between 20 and 25 mg, were homogenized in 100 μL of either NAD or NADH extraction buffer. The extracts were subsequently heated at 60°C for 5 min. Following this, 20 μL of assay buffer and 100 μL of the corresponding opposite extraction buffer were sequentially added. The samples underwent vortexing and were centrifuged at 14,000 rpm for 5 min. The resulting clear supernatant was collected for the assay. For each well, 40 μL of sample or standard and 80 μL of working reagent were added. Absorbance was measured at 0 and 15-min intervals at an optical density of 565 nm at room temperature. The ratio was calculated using the manufacturer’s equation, and the NAD + content was normalized to the protein concentration.

### Oil red O staining

The hepatic tissue was initially fixed in 4% paraformaldehyde for 4 h following dissection into small pieces. Subsequently, the tissue was embedded in OCT (Leica Camera AG, Wetzlar, Germany) and frozen sections with a thickness of 8 µm were obtained using a cryostat. These sections underwent an additional 30-minute fixation in 4% paraformaldehyde before being rinsed in distilled water and stained with Oil Red O for 15 min. Hematoxylin was used as a counterstain for 10 s to visualize nuclei on the slides. Ultimately, the oil red staining results were measured using ImageJ.

### RNA-seq and data processing

Total RNA was isolated from tissues using Trizol reagent. mRNA was purified using oligo (dT)-attached magnetic beads. The purified mRNA was fragmented using fragment buffer at the appropriate temperature. First-strand cDNA was synthesized by random hexamer-primed reverse transcription, followed by second-strand cDNA synthesis. Subsequent steps involved addition of A-Tailing Mix and RNA Index Adapters through incubation for end repair. The cDNA fragments obtained previously were amplified by PCR, and the resulting products were purified using Ampure XP Beads before dissolving in elution buffer. Quality control was performed by validating the product on a 2100 bioanalyzer (Agilent Technologies, Santa Clara, CA, USA). Double-stranded PCR products were subjected to heating, denaturation, and circularization using the splint oligo sequence to obtain the final library. The final library as single-stranded circular DNA was further amplified using phi29 to generate DNA nanoballs, each containing >300 copies of a single molecule. These DNA nanoballs were loaded onto a patterned nanoarray, and paired-end 50-base pair reads were generated using the BGIseq500 platform (BGI, Shenzhen, China).

Sequencing data underwent filtering with SOAPnuke (v1.5.2) (Li et al., 2008), which involved removal of reads containing sequencing adapters, elimination of reads with a low-quality base ratio (≤5 base quality) exceeding 20, and exclusion of reads with an unknown base (Nʹ base) ratio exceeding 5%. This process yielded clean reads stored in FASTQ format that were mapped to the reference genome using HISAT2 (v2.0.4) (Kim et al., 2019). Bowtie2 (v2.2.5) (Langdon, 2015) was employed to align the clean reads with the reference coding gene set, following which gene expression levels were calculated using RSEM (v1.2.12) (Li and Dewey, 2011). The resulting gene expression data were used to generate a heatmap through pheatmap (v1.0.8) (Kolde and Kolde, 2015), illustrating gene expression variations across samples. Differential expression analysis was conducted using DESeq2 (v1.4.5) (Love et al., 2014) with a significance threshold of Q < 0.05. To gain insights into phenotypic changes, GO (http://www.geneontology.org/) and KEGG (Kanehisa and Goto, 2000) (https://www.kegg.jp/) enrichment analyses were performed on annotated differentially expressed genes using Phyper (https://en.wikipedia.org/wiki/Hypergeometric distribution) based on the hypergeometric test. The significance level of terms and pathways was adjusted using the Bonferroni test with a stringent threshold of <0.05.

### Untargeted metabolomics and LC-MS/MS analysis

A sample (50 mg) was placed in an Eppendorf tube, and 1 mL extract solution (methanol:acetonitrile:water = 2:2:1 with an isotopically labeled internal standard mixture) was added. The sample was homogenized at 35 Hz for 4 min and sonicated for 5 min in an iced water bath. The homogenization and sonication cycles were repeated three times. The sample was incubated for 1 h at −40°C and then centrifuged at 12,000 g for 15 min at 4°C. The resulting supernatant was transferred to a fresh glass vial for analysis.

LC-MS/MS analyses were performed using a UHPLC system (Vanquish; Thermo Fisher Scientific) with a UPLC BEH Amide column (2.1 mm × 100 mm, 1.7 μm) coupled to a Q Exactive HFX mass spectrometer (Orbitrap MS; Thermo Fisher Scientific). The mobile phases consisted of 25 mmol/L ammonium acetate and 25 mmol/L ammonia hydroxide in water (pH 9.75) (A) or acetonitrile (B). The auto-sampler temperature was 4°C, and the injection volume was 2 μL. The QE HFX mass spectrometer was used to acquire MS/MS spectra using the information-dependent acquisition mode with acquisition software (Xcalibur; Thermo Fisher Scientific). In this mode, the acquisition software continuously evaluated the full MS spectrum. Emergency severity index source conditions were set as follows: sheath gas flow rate, 30 Arb; Aux gas flow rate. 25 Arb; capillary temperature. 350°C; full MS resolution, 60,000; MS/MS resolution, 7500; collision energy, 10/30/60 in normalized collisional energy mode; spray voltage, 3.6 kV (positive) or −3.2 kV (negative).

Raw data were converted to the mzXML format using ProteoWizard and processed with an in-house program developed using R and based on XCMS for peak detection, extraction, alignment, and integration. An in-house MS2 database (BiotreeDB) was used for metabolite annotation. The cutoff for annotation was set at 0.3. The data utilized for the analysis are presented in Supplementary Table S2.

### Weighted gene Co-expression network analysis

The Dr Tom Multi-Omics Data Mining system (https://biosys.bgi.com) from BGI company (Shenzhen, China) was used for WGCNA analysis.

### Western blot

Western blot analyses were conducted using SDS-PAGE. Tissue cells were lysed in RIPA buffer with protease inhibitors (Complete Mini Protease Inhibitor cocktail tablets, Roche) and PMSF, then centrifuged to obtain soluble proteins. Protein concentration was measured via BCA. Equal protein amounts were separated by SDS-PAGE and transferred to nitrocellulose membranes. Membranes were incubated with primary antibodies overnight at 4°C, followed by secondary antibodies for 1–2 h. Antibodies used included anti-GAPDH at 1:5000 (Proteintech, 10494-1-AP), anti-SIRT1 at 1:5000 (Proteintech, 60303-1-Ig), anti-P53 at 1:500 (Abcam, ab26), and anti-p16INK4a at 1:500 (Proteintech, 30519-1-AP). Bands were visualized using chemiluminescence with an ECL detection kit (Clarity Western ECL Substrate, Bio-Rad).

### SIRT1 activity assay

To evaluate cellular SIRT1 activity, proteins were isolated from hepatic tissue cells. The SIRT1 Fluorometric Assay Kit (Elabscience, E-BC-F056) was employed to quantify SIRT1 activity following the manufacturer’s protocol. Fluorescence intensities were subsequently measured using a microplate fluorometer, with an excitation wavelength of 340 nm and an emission wavelength of 440 nm.

### Open field test (OFT)

To assess the mouse anxiety level, we used a square box measuring 50 × 50 × 30 cm. Anxious mice tended to favor the box corners and exhibited stereotypical movements along the box sides. Mice were placed in a designated box corner and their movements in the box were tracked and recorded for 20 min. We gauged mouse anxiety by observing the amount of time spent in the central area.

### Novel object recognition (NOR) test

NOR tests evaluate the natural inclination of rodents to approach and explore unfamiliar objects, serving as a behavioral test to assess their recognition memory. The NOR test includes three distinct phases: adaptation, familiarization, and test phases. Adaptation phase: During the adaptation period, a square black box was introduced as the new object. Mice were placed in one corner of the black box and allowed to freely explore the box for 10 min. This adaptation phase acclimatized the mice to the unfamiliar environment and reduced their stress level. Familiarization phase: Two identical objects (A1 and A2) were positioned diagonally across the bottom of the black box, maintaining a specific distance from the sides. Mice were introduced to one corner of the black box and given the freedom to explore and become acquainted with these objects for 10 min. Subsequent to the familiarization phase, the mice were returned to their cages for additional familiarization and later subjected to testing after a predetermined interval. Test phase: A1 was replaced with a new object denoted as B. Mice were reintroduced to the same corner of the black box, where they were initially placed during adaptation and familiarization phases. They were then allowed to freely explore the new object for 5 min. Video recordings were employed to monitor mouse exploration times of A2 and B. The preference for the new object was quantified using the discrimination index denoted as D2 and calculated by the formula: D2 = N/(FN + F), where N is the exploration time dedicated to B, FN is the exploration time directed towards B, F is the exploration time dedicated to A2.

### Statistical analysis

All data are expressed as the mean ± standard error of the mean (SEM). Statistical significance was assessed using a two-tailed Student’s t-test or a one-way analysis of variance (ANOVA), as specified in the figure legends. In cases where ANOVA indicated a significant difference among groups, pairwise comparisons were conducted with the Bonferroni correction for multiple comparisons. Statistical significance was defined at three levels: *p < 0.05, **p < 0.01, and ***p < 0.001. Mice that either died before the conclusion of the experiment, exhibited illness, or were identified as statistical outliers (as determined by Grubb’s outlier test) were excluded from the final analysis.

## Results

### Beneficial effect of PncA overexpression on hepatic and renal NAD^+^ levels and age-related changes

**FIGURE 1:**
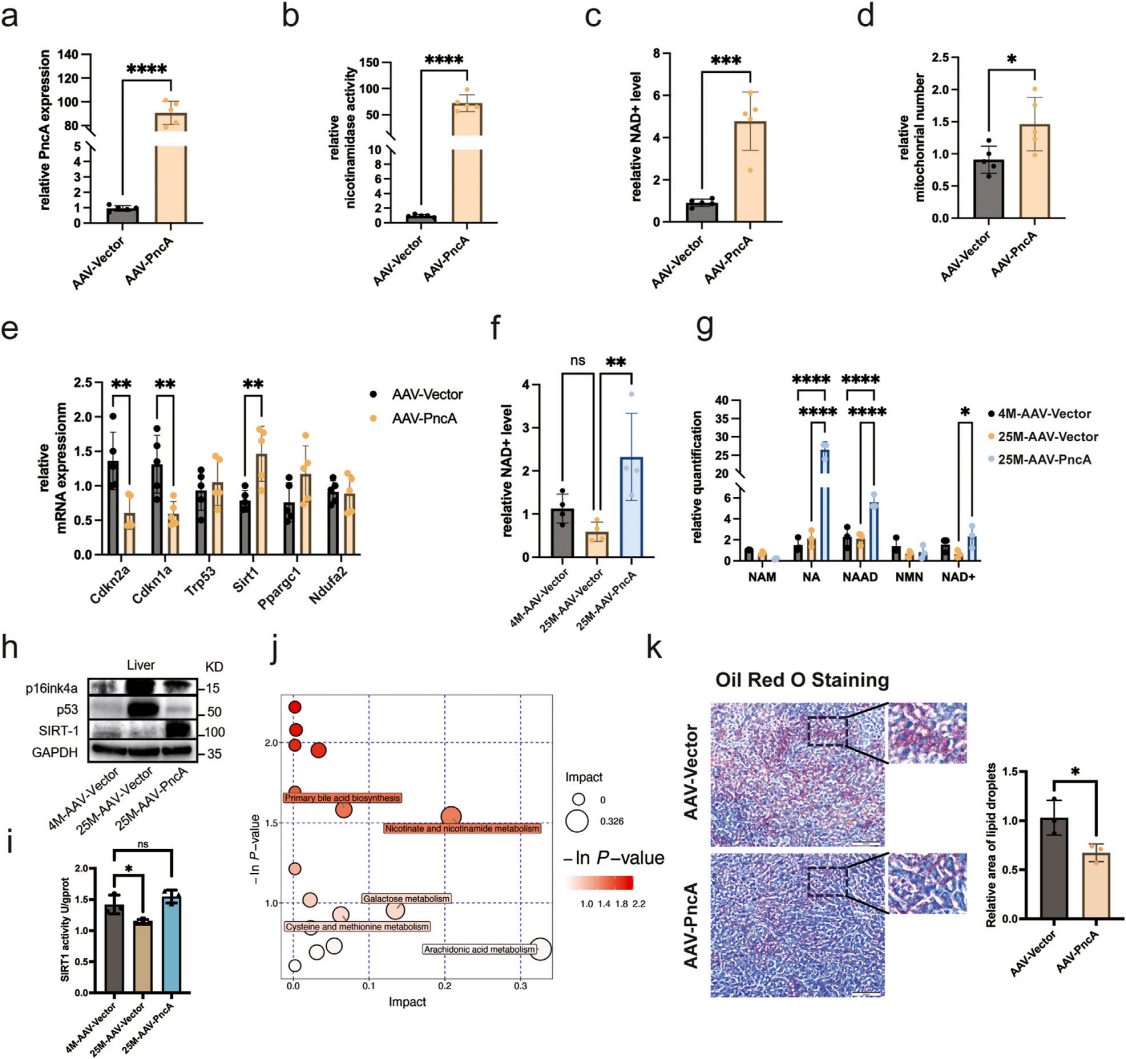
PncA Increases the NAD^+^ Level and Alleviates Liver Aging **(a)** Expression level of the *PncA* gene in the liver after AAV-PncA infection (n = 5 for each group, ****p < 0.0001) **(b)** Identification of nicotinamide activity in homogenized liver tissue samples (n = 5 for each group, ****p < 0.0001) **(c)** Measurement of NAD^+^ levels following PncA overexpression in middle-aged mice (n = 5 for each group, ***p < 0.001) **(d)** qPCR-based mitochondrial quantification (n = 5 for each group, *p < 0.05) **(e)** Expression levels of genes associated with aging and mitochondrial function (n = 5 for each group, **p < 0.01) **(f)** NAD^+^ levels in young mice with vector expression, aged mice with vector expression, and aged mice with PncA overexpression (n = 5 for young and aged mice with *PncA* overexpression group, n = 4 for aged mice group **p < 0.01) **(g)** Levels of NAD^+^-related metabolites in non-targeted metabolomic data (n = 3 for each group. ****p < 0.0001, **p < 0.01) **(h)** Western blot analysis of p16^Ink4a^, p53, and SIRT1 proteins linked to aging and mitochondrial function in aged mouse livers with *PncA* overexpression **(i)** Effect of *PncA* overexpression on SIRT1 protein activity (n = 3 for each group, *p < 0.05) **(j)** Major metabolic pathways enriched with metabolites affected by PncA in non-targeted metabolomic data **(k)** Analysis of oil red staining in the liver and the statistical outcomes related to lipid droplets. Scale bars 500 µm.

We engineered an adeno-associated virus (AAV) 9 specifically for overexpression of *PncA*. This virus was administered intravenously to middle-aged mice (8 months old), targeting tissues, including the heart, liver, kidneys, and brain. Our analyses revealed pronounced upregulation of *PncA* in the liver (Figure 1A), which was accompanied by a notable increase in deamidase activity in liver lysates (Figure 1B), aligning with our previous findings (Feng et al., 2023). Importantly, *PncA* overexpression resulted in a substantial elevation of the hepatic NAD^+^ level in middle-aged mice (Figure 1C). Considering the pivotal role of mitochondria in NAD^+^ metabolism, we quantified mitochondrial content in the liver and observed a marked increase after *PncA* overexpression (Figure 1D). We also evaluated the expression of major aging-related genes, noting significant downregulation of *Cdkn2a* (encoding p16^Ink4a^) and *Cdkn1a* (encoding p21), and upregulation of genes critical for mitochondrial functions, such as *Sirt1* and *Ppargc1* (Figure 1E).

To address the connection between NAD^+^ and aging, we extended our investigation to older mice (25 months old). Remarkably, *PncA* overexpression elevated the hepatic NAD^+^ level in older mice to above that in younger counterparts (Figure 1F). Metabolomic profiling data analysis revealed higher concentrations of NA and its downstream product, NAAD, following *PncA* overexpression. The changes in NAD^+^ levels corroborated our NAD^+^ assay findings (Figure 1G). Given that *PncA* overexpression has been shown to elevate NAD^+^ levels in aged mice, we subsequently assessed the protein expression of the senescence-associated marker p16^Ink4a^ and p53 and the mitochondrial function-related protein SIRT1 using Western blot analysis (Figure 1H; Supplementary Figure S1A). Moreover, the detection of SIRT1 protein activity revealed that PncA could enhance SIRT1 protein activity in the liver tissue cells of aged mice (Figure 1I). The results demonstrated that *PncA* overexpression significantly attenuated hepatocyte senescence and enhanced mitochondrial function. Moreover, our findings indicated that although aging is typically associated with heightened inflammatory pathways in the liver, the overexpression of *PncA* resulted in a significant suppression of certain inflammatory pathways in aged mice (Supplementary Figure S1B).

Metabolic pathway enrichment analysis highlighted *PncA*’s influence on specific pathways, including nicotinate and nicotinamide metabolism, arachidonic acid metabolism, and primary bile acid biosynthesis (Figure 1J). With increasing age, the liver’s metabolic function and ability to maintain fat balance may decline (He et al., 2023). This includes a reduced capacity for fatty acid oxidation and synthesis, and ability to synthesize and excrete bile acids. These changes lead to abnormal accumulation of fatty acids in the liver, resulting in a fatty liver. Our results suggested that *PncA* overexpression may counteract the age-related decline in hepatic metabolic and fat-balancing capabilities, potentially mitigating fatty liver development in aged mice (Figure 1K).

**FIGURE 2:**
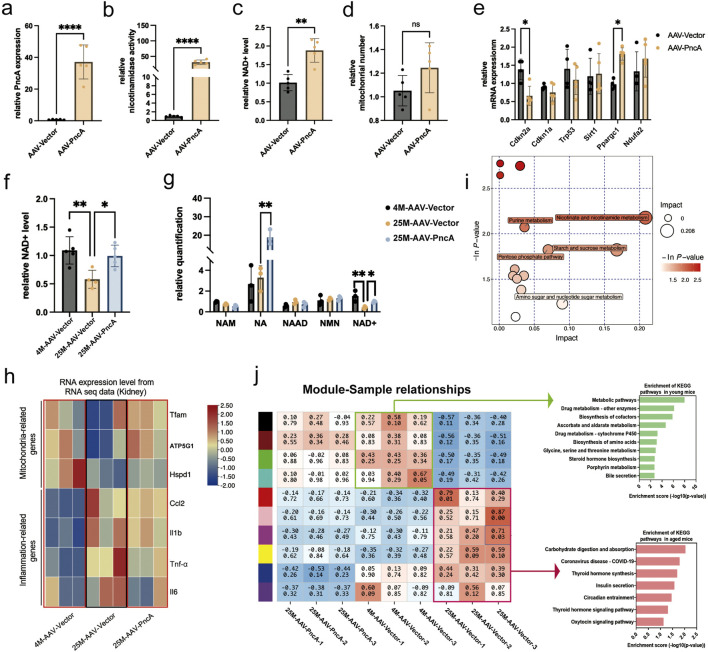
PncA Increases the Kidney NAD^+^ Level and Alleviates Kidney Aging **(a)** Expression level of the PncA gene in kidneys after AAV-PncA infection (n = 5 for each group, ****p < 0.0001) **(b)** Identification of nicotinamide activity in homogenized kidney tissue samples (n = 5 for each group, ****p < 0.0001) **(c)** Measurement of NAD^+^ levels following *PncA* overexpression in middle-aged mice (n = 5 for each group, **p < 0.01) **(d)** qPCR-based mitochondrial quantification (n = 5 for each group) **(e)** Expression levels of genes associated with aging and mitochondrial function (n = 5 for each group, *p < 0.05) **(f)** NAD^+^ levels in young mice with vector expression, aged mice with vector expression, and aged mice with *PncA* overexpression (n = 5 for young and aged mice with *PncA* overexpression group, n = 4 for aged mice group, *p < 0.05, **p < 0.01) **(g)** Levels of NAD^+^-related metabolites in non-targeted metabolomic data (n = 3 for each group. *p < 0.05, **p < 0.01) **(h)** The impact of increased *PncA* expression on genes associated with mitochondrial function and inflammatory processes (n = 3 for each group) **(i)** Major metabolic pathways enriched with metabolites affected by PncA in non-targeted metabolomic data **(j)** WGCNA and KEGG pathway analysis of gene expression in the kidneys of young mice with vector expression, aged mice with vector expression, and aged mice with PncA overexpression.

We observed outcomes of mouse kidneys that closely paralleled those observed in the liver. As depicted in Figures 2A, B, we achieved successful overexpression of *PncA* in kidneys. Furthermore, we noted significant augmentation of the NAD^+^ level and mitochondrial quantity in kidneys. However, only the change in NAD^+^ levels showed statistical significance (Figures 2C, D). These findings suggested that, similar to the liver, kidneys exhibited a propensity to favor the use of NA rather than NAM for NAD^+^ synthesis. Additionally, our evaluation of aging-related and essential mitochondrial function genes indicated a marked decrease in p16^Ink4a^ expression and a marked increase in *Ppargc1a* expression (Figure 2E). In a manner analogous to the liver, we explored whether *PncA* overexpression mitigated the aging process in the kidneys of elderly mice. Remarkably, the NAD^+^ level was reinstated to that observed in young mice following *PncA* overexpression (Figure 2F). Metabolomic profiling data revealed a marked increase in NA and NAD^+^ levels in accordance with the NAD^+^ level quantified by assay kits (Figure 2G). Like with the liver, we assessed its anti-aging impact by examining how *PncA* overexpression affects the kidneys in aged mice. The findings indicate that *PncA* overexpression significantly upregulated the expression of genes associated with mitochondrial function in aged renal cells, while it partially downregulated the expression of genes related to inflammation (Figure 2H).

Metabolomic profiling data indicated that *PncA* primarily influenced pathways associated with nicotinate and nicotinamide metabolism, purine metabolism, and the pentose phosphate pathway (Figure 2I). Furthermore, we determined the expression profiles of young and aged mice, followed by modular clustering analysis employing weighted gene co-expression network analysis (WGCNA) (Langfelder and Horvath, 2008). Our findings revealed that, in the kidneys of aged mice, *PncA* overexpression led to an expression profile that more closely resembled that of the young group. Among these, the primary manifestation is the enhancement of metabolic pathways in the kidneys of aged mice (Figure 2J).

This compelling observation strongly implied that *PncA* mitigated the renal aging process in elderly mice. Moreover, the genes were isolated from the modules identified by WGCNA. Subsequent heatmap analysis indicated that specific genes exhibited upregulation in older age, yet their expression was downregulated following *PncA* overexpression. Conversely, certain genes that were downregulated in elderly individuals were reactivated upon *PncA* overexpression (Supplementary Figure S1C). Enrichment analysis demonstrated that these genes were predominantly enriched in pathways linked to aging, including the PI3K-Akt signaling pathway and MAPK signaling pathway (Supplementary Figure S1D). This finding reinforces the hypothesis that *PncA* is involved in regulating the aging process, specifically in the renal system of aged mice.

### Detrimental effects of *PncA* overexpression on cardiac and hippocampal NAD^+^ levels and age-associated alterations

**FIGURE 3:**
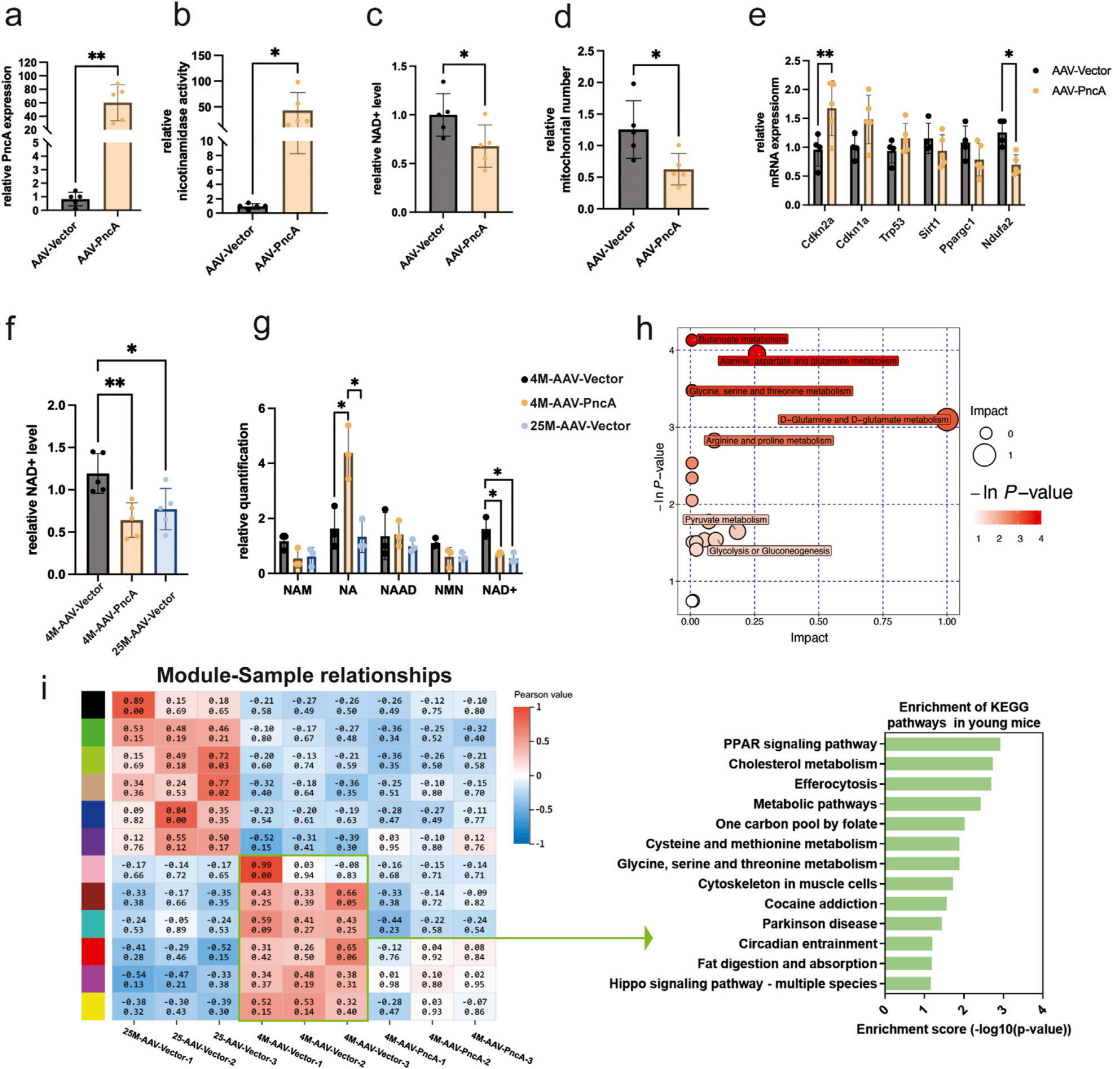
PncA reduces the cardiac NAD^+^ level and promotes aging in the young mouse cardiac cells **(a)** Expression level of the *PncA* gene in the heart after AAV-PncA infection (n = 5 for each group, **p < 0.01) **(b)** Identification of nicotinamide activity in homogenized heart tissue samples (n = 5 for each group, *p < 0.05) **(c)** Measurement of NAD^+^ levels following *PncA* overexpression in middle-aged mice (n = 5 for each group, *p < 0.05) **(d)** qPCR-based mitochondrial quantification (n = 5 for each group, *p < 0.05) **(e)** Expression levels of genes associated with aging and mitochondrial function (n = 5 for each group, *p < 0.05, **p < 0.01) **(f)** NAD^+^ levels in young mice with vector expression, aged mice with vector expression, and young mice with *PncA* overexpression (n = 5 for each group, *p < 0.05, **p < 0.01) **(g)** Levels of NAD^+^-related metabolites in non-targeted metabolomic data (n = 3 for each group. *p < 0.05) **(h)** Major metabolic pathways enriched with metabolites affected by *PncA* in non-targeted metabolomic data **(i)** WGCNA and KEGG pathway analysis of gene expression in the hearts of young mice with vector expression, aged mice with vector expression, and young mice with *PncA* overexpression.

Contrasting outcomes were observed in the hearts and hippocampi of mice. Initially, we achieved significant expression of the *PncA* gene in the hearts of 8-month-old mice through AAV9 delivery (Figure 3A), and enzymatic activity assays using heart homogenates revealed substantial deamidase activity (Figure 3B). Nonetheless, a notable decrease was found in the NAD^+^ level and mitochondrial count in the *PncA* group (Figures 3C, D). This indicates that, unlike the liver and kidney, the heart shows a preference for utilizing NAM over NA in the biosynthesis of NAD^+^. Simultaneously, we conducted an analysis of the effects of *PncA* overexpression in cardiac tissue on genes associated with aging and mitochondrial function. Our findings revealed upregulation of aging marker p16^Ink4a^ and downregulation of *Ndufa2*, a gene related to mitochondrial functions (Figure 3E). Considering the adverse effect of *PncA* on the cardiac NAD^+^ level, we overexpressed the *PncA* gene in young 4-month-old mice to investigate its effects on the cardiac NAD^+^ level and aging process in youthful hearts. Remarkably, the NAD^+^ level exhibited a significant decline in the *PncA* group, reaching a level close to that observed in 25-month-old mice (Figure 3F). Metabolomic profiling data corroborated changes in NA and NAD^+^ levels, which aligned with our expectations (Figure 3G). Collectively, these findings imply that *PncA* exerts a negative influence on the cardiac function in young mice.

Enrichment analysis of metabolites influenced by *PncA* in metabolomic profiling data revealed strong effects primarily on D-glutamine and D-glutamate, arginine and proline, and pyruvate metabolisms (Figure 3H). Similarly, we conducted RNA sequencing of mouse hearts in the various groups and WGCNA, as described above for kidney tissue. The findings indicated that *PncA* overexpression in the young mouse group led to the suppression of numerous genes that are typically expressed in this group, resulting in the loss of their youthful mode of gene expression (Figure 3I). A KEGG pathway analysis of genes enriched in the hearts of these young mice and influenced by *PncA* overexpression revealed significant impacts on the PPAR signaling pathway, cholesterol metabolism, and efferocytosis pathways. These alterations contributed to the premature aging of the cardiac tissue cell in the young mice.

**FIGURE 4:**
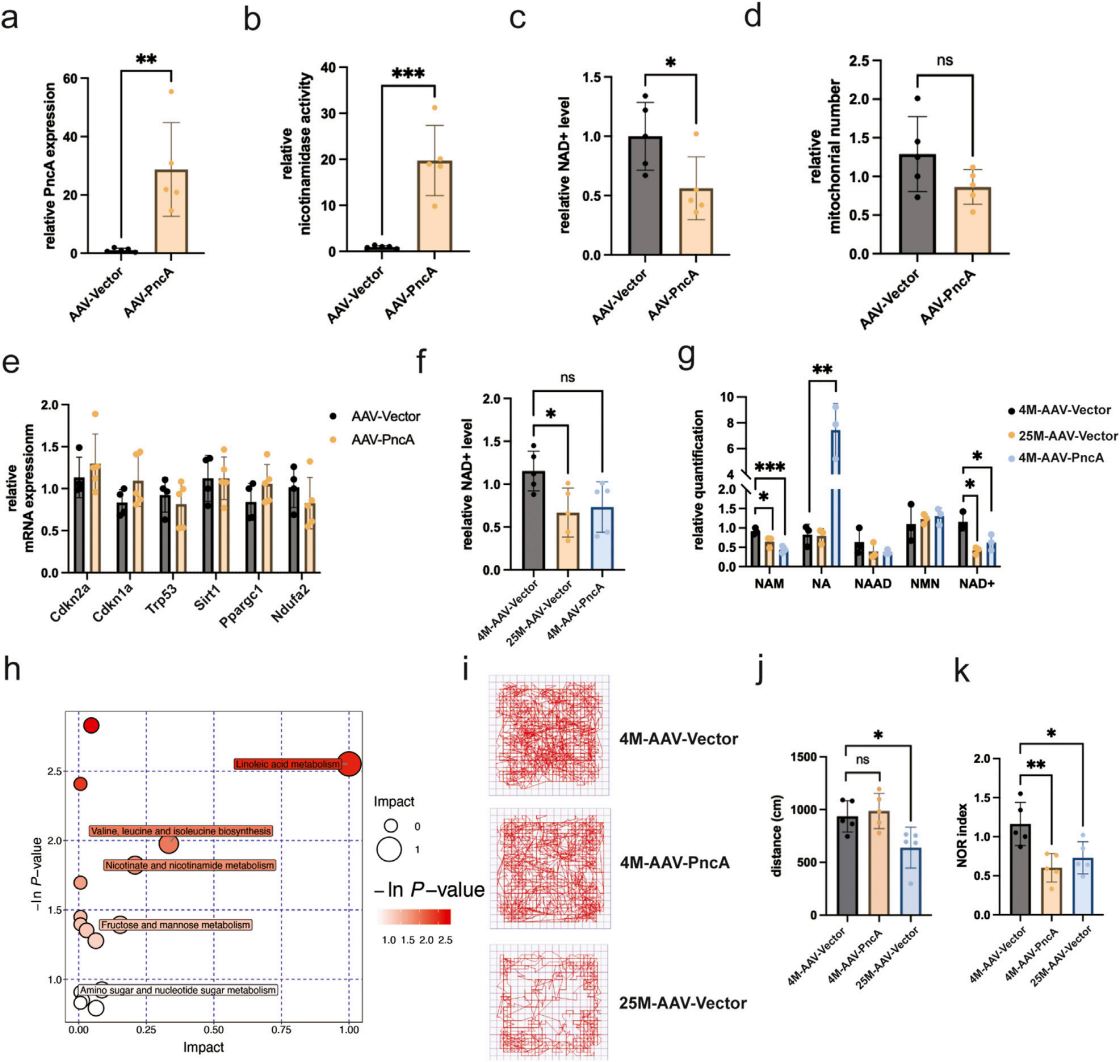
PncA reduces the hippocampal NAD^+^ level and impairs cognitive function in young mice **(a)** Expression level of the *PncA* gene in the hippocampus after AAV-PncA infection (n = 5 for each group, **p < 0.01) **(b)** Identification of nicotinamide activity in homogenized hippocampus tissue samples (n = 5 for each group, ***p < 0.001) **(c)** Measurement of NAD^+^ levels following *PncA* overexpression in middle-aged mice (n = 5 for each group, *p < 0.05) **(d)** qPCR-based mitochondrial quantification (n = 5 for each group) **(e)** Expression levels of genes associated with aging and mitochondrial function **(f)** NAD^+^ levels in young mice with vector expression, aged mice with vector expression, and young mice with *PncA* overexpression (n = 5 for each group, *p < 0.05) **(g)** Analysis of NAD^+^-related metabolite levels in non-targeted metabolomic data from hippocampal tissue. (n = 3 for each group, *p < 0.05, **p < 0.01, ***p < 0.001) **(h)** Major metabolic pathways enriched with metabolites affected by *PncA* in non-targeted metabolomic data **(i)** Trajectories of mice tracked by software in the OFT **(j)** The travel distance bar graph of the mice in the OFT (n = 5 for each group, *p < 0.05) **(k)** Bar graph of cognitive index statistics of mice (n = 5 for each group, *p < 0.05, **p < 0.01).

Results analogous to those observed in the heart were noted in the hippocampi of mouse brains. The successful overexpression of *PncA* in the mouse hippocampus was confirmed through Figures 4A, B. A significant reduction in both NAD^+^ levels and mitochondrial count in the hippocampus was observed, with statistical significance only found in the change in NAD^+^ levels (Figures 4C, D). This observation underscored that, similar to the heart, the hippocampus exhibited preferential use of NAM rather than NA for NAD^+^ synthesis. However, no statistically significant alterations were found in the expression of associated aging marker genes following *PncA* overexpression (Figure 4E). Next, we explored whether *PncA* overexpression prompted the aging process in the hippocampi of young mice. Although the NAD^+^ level declined to a level like that in elderly mice after *PncA* overexpression, statistical significance was not reached (Figure 4F). Metabolomic profiling data revealed a noteworthy decline in the NAM level in the aging hippocampus. After *PncA* overexpression, the NA level in the hippocampus was increased significantly, which was congruent with the NAD^+^ level measured by assay kits (Figure 4G). Metabolomic profiling data suggested that *PncA* primarily affected metabolic pathways in the hippocampus, encompassing valine, leucine, and isoleucine biosynthesis, nicotinate and nicotinamide metabolism, and linoleic acid metabolism (Figure 4H). Previous studies have underscored the pivotal role of the NAD^+^ level in the hippocampus in influencing mouse cognitive functions (Zhao et al., 2021; Hou et al., 2018). Considering the adverse effect of *PncA* on the NAD^+^ level in the hippocampus of young mice, we investigated alterations in mouse cognitive functions by open field and novel object recognition tests. The results revealed that *PncA* overexpression exerted no discernible influence on the movement trajectories of young mice in the open field test (Figure 4I), but the trajectories of aged mice were notably reduced (Figure 4J), and they exhibited a preference for the four box corners (Figure 4I). Conversely, elderly mice exhibited fewer trajectories that were more concentrated in the four box corners compared with young mice. However, in the novel object recognition test, both elderly and young mice in the *PncA* overexpression group demonstrated a pronounced decline in cognitive functions relative to normal young mice (Figure 4K). These findings indicate that *PncA* advances the aging process in the mouse hippocampus.

## Discussion

In this comprehensive investigation of NAD^+^ metabolism and its implications in aging, we obtained valuable insights into the tissue-specific dynamics of NAD^+^ synthesis and its effects on the aging process. It is well-documented that NAD^+^ levels decline with aging, thereby exacerbating neurodegenerative diseases—such as energy deficiency, abnormal protein aggregation, and neuroinflammation—as well as metabolic disorders, including insulin resistance, lipid accumulation, and chronic inflammation. These conditions are influenced by the impact of NAD^+^ on mitochondrial function, deacetylase activity, autophagy, and inflammation. While supplementation with NAD^+^ precursors, such as nicotinamide mononucleotide (NMN), or interventions targeting NAD^+^ synthesis and depletion pathways hold potential for ameliorating these diseases, significant challenges remain. These include the need for effective tissue targeting and the assessment of long-term safety.

Our study focused on modulation of the NAD^+^ level through overexpression of nicotinamidase *PncA* in various mouse tissues, shedding light on the intricate interplay between NAD^+^ metabolism and aging across various tissues.

Our results demonstrate that the effect of *PncA* on the NAD^+^ level is specific to the tissue. *PncA* overexpression elevated the NAD^+^ level and delayed aging marker expression in the liver and kidneys, but it had contrasting effects on the cardiac cells and hippocampus, where it accelerated aging and resulted in cognitive decline. The results highlight the variability in the utilization efficiency of NAD^+^ precursors across various tissues, with the liver and kidneys demonstrating a preference for NA in NAD^+^ synthesis, while the heart and hippocampus exhibit a preference for NAM in NAD^+^ production. These findings underscore the importance of adopting a tissue-specific strategy when contemplating NAD^+^ precursor supplementation for anti-aging interventions. Moreover, our study is in accordance with previous research on the diverse impacts of NAD^+^ on mitochondrial function and cellular senescence (Babbar et al., 2020). This equilibrium is disrupted in the aging process and in several age-related illnesses, as elucidated by Yang et al. In their study, supplementation of NAD^+^ was found to ameliorate senescence characteristics and enhance motor function in Atm^−/−^ mice, presenting a novel viewpoint on the involvement of NAD^+^ in the aging brain (Yang et al., 2021).

The most important aspect is that our study introduces a pioneering technique to modify mammalian NAD^+^ metabolism, focusing on the distinct preferences for NAD^+^ precursors among tissues. Through overexpression of *PncA*, this study simulated NAD^+^ precursor conversions in various tissues and distinctly mapped the efficiency of these conversions for NAD^+^ synthesis. This innovative method overcomes the typical constraints in conventional NAD^+^ precursor supplementation, such as variable absorption efficiencies and microbiota-induced metabolic alterations, thereby profoundly enhancing our comprehension of NAD^+^ metabolic pathways and laying the groundwork for tissue-specific aging intervention strategies.

One significant constraint of our present study is the absence of direct examination into the various factors contributing to differential NAD^+^ consumption, such as oxidative stress, DNA damage, and changes in PARylation activities. These elements play a crucial role in elucidating the tissue-specific intricacies of NAD^+^ metabolism, impacting fundamental cellular functions like mitochondrial activity and the cellular reaction to harm. Our research highlights a crucial area for further study: understanding the molecular mechanisms that underlie these metabolic variations. This exploration may provide valuable information for the development of targeted strategies for NAD^+^ supplementation, leading to innovative approaches for managing aging and age-related illnesses.

## Data Availability

The RNA-seq raw sequencing data files generated in this study are available in the NCBI’s Sequence Read Archive (SRA) under BioProject accession number PRJNA PRJNA1070374.

## Supplementary material

Supplementary Figure S1 PncA ameliorates the gene expression profile in the liver and kidneys during the aging process. a. Expression level of genes associated with mitochondrial function (n = 5 for young and aged mice with *PncA* overexpression group, n = 4 for aged mice group *p < 0.05, **p < 0.01, ****p < 0.0001). b. Expression level of genes associated with inflammation (n = 5 for young and aged mice with *PncA* overexpression group, n = 4 for aged mice group *p < 0.05, ***p < 0.001). c. Heatmap of gene expression within modules identified by WGCNA analysis. d. KEGG enrichment analysis of genes in heatmap.
