# Supplementary material for: Tissue-specific effects of bacterial PncA overexpression on NAD+ metabolism and aging in mice: implications for tissue-specific aging interventions
Source: Front Aging. 2025 Apr 28;6:1546017. doi: 10.3389/fragi.2025.1546017 (PMC12066511; doi:10.3389/fragi.2025.1546017)
Supplement: Supplementary file 2 [file Table1.docx]

**Supplementary Table 1. Primer sequences used in this study.**

| **Target gene** | | **Forward primer (5'--3')** | **Reverse primer (5'--3')** |
| --- | --- | --- | --- |
|  | Cdkn2a | CGCAGGTTCTTGGTCACTGT | TGTTCACGAAAGCCAGAGCG |
|  | Cdkn1a | CCTGGTGATGTCCGACCTG | CCATGAGCGCATCGCAATC |
|  | Trp53 | GTCACAGCACATGACGGAGG | TCTTCCAGATGCTCGGGATAC |
|  | Sirt1 | TCGTGGAGACATTTTTAATCAGG | GCTTCATGATGGCAAGTGG |
|  | Ppargc1 | TATGGAGTGACATAGAGTGTGCT | CCACTTCAATCCACCCAGAAAG |
|  | Nduf2a | TGGTCCGATTGAGGGTGAAAG | GGGAACGCTGGCATAAGTGA |
|  | PncA | TGATCGCCAGCCAAGACT | AGCATCCAGCACCGTGAA |
|  | Il6 | TAGTCCTTCCTACCCCAATTTCC | TTGGTCCTTAGCCACTCCTTC |
|  | TNF-α | GGTGCCTATGTCTCAGCCTCTT | GCCATAGAACTGATGAGAGGGAG |
|  | Il1β | GCAACTGTTCCTGAACTCAACT | ATCTTTTGGGGTCCGTCAACT |
|  | Ccl2 | TTAAAAACCTGGATCGGAACCAA | GCATTAGCTTCAGATTTACGGGT |
|  | Tfam | ATTCCGAAGTGTTTTTCCAGCA | TCTGAAAGTTTTGCATCTGGGT |
|  | Cs | GGACAATTTTCCAACCAATCTGC | TCGGTTCATTCCCTCTGCATA |
|  | ATP5G1 | CCAGAGGCCCCATCTAAGC | CCCCAGAATGGCATAGGAGAAG |
|  | Hspd1 | CACAGTCCTTCGCCAGATGAG | CTACACCTTGAAGCATTAAGGCT |
